# Supplementary material for: Predicting physiologically-relevant oxygen concentrations in precision-cut liver slices using mathematical modelling
Source: PLoS One. 2022 Nov 2;17(11):e0275788. doi: 10.1371/journal.pone.0275788 (PMC9629643; doi:10.1371/journal.pone.0275788)
Supplement: S1 Data — (PDF) [file pone.0275788.s001.pdf]

Minimal Data Set

This document details the parameter values used in the paper "Predicting physiologically-relevant oxygen concentrations in precision-cut liver slices using mathematical modelling" to produce figures 2-7.

The parameters values contained in the table below are used in every simulation in the paper

Figure 2

a) $\phi_0 = 160$, $h_1 = 3.149 \times 10^{-3}$, $h_2 = 3.399 \times 10^{-3}$, $r_T = 2.5 \times 10^{-3}$

b) $\phi_0 = 160$, $h_1 = 3.149 \times 10^{-3}$, $h_2 = 3.399 \times 10^{-3}$, $r_T = 4 \times 10^{-3}$

c) $\phi_0 = 160$, $h_1 = 1.575 \times 10^{-3}$, $h_2 = 1.825 \times 10^{-3}$, $r_T = 2.5 \times 10^{-3}$

d) $\phi_0 = 160$, $h_1 = 1.575 \times 10^{-3}$, $h_2 = 1.825 \times 10^{-3}$, $r_T = 4 \times 10^{-3}$

e) $\phi_0 = 160$, $h_1 = 0$, $h_2 = 2.5 \times 10^{-4}$, $r_T = 2.5 \times 10^{-3}$

f) $\phi_0 = 160$, $h_1 = 0$, $h_2 = 2.5 \times 10^{-4}$, $r_T = 4 \times 10^{-3}$

Figure 3

a) $\phi_0 = 609.524$, $h_1 = 3.149 \times 10^{-3}$, $h_2 = 3.399 \times 10^{-3}$, $r_T = 2.5 \times 10^{-3}$

b) $\phi_0 = 609.524$, $h_1 = 3.149 \times 10^{-3}$, $h_2 = 3.399 \times 10^{-3}$, $r_T = 4 \times 10^{-3}$

c) $\phi_0 = 609.524$, $h_1 = 1.575 \times 10^{-3}$, $h_2 = 1.825 \times 10^{-3}$, $r_T = 2.5 \times 10^{-3}$

d) $\phi_0 = 609.524$, $h_1 = 1.575 \times 10^{-3}$, $h_2 = 1.825 \times 10^{-3}$, $r_T = 4 \times 10^{-3}$

e) $\phi_0 = 609.524$, $h_1 = 0$, $h_2 = 2.5 \times 10^{-4}$, $r_T = 2.5 \times 10^{-3}$

f) $\phi_0 = 609.524$, $h_1 = 0$, $h_2 = 2.5 \times 10^{-4}$, $r_T = 4 \times 10^{-3}$

Figure 4

a) $\phi_0 = 723.810$, $h_1 = 3.149 \times 10^{-3}$, $h_2 = 3.399 \times 10^{-3}$, $r_T = 2.5 \times 10^{-3}$

b) $\phi_0 = 723.810$, $h_1 = 3.149 \times 10^{-3}$, $h_2 = 3.399 \times 10^{-3}$, $r_T = 4 \times 10^{-3}$

c) $\phi_0 = 723.810$, $h_1 = 1.575 \times 10^{-3}$, $h_2 = 1.825 \times 10^{-3}$, $r_T = 2.5 \times 10^{-3}$

d) $\phi_0 = 723.810$, $h_1 = 1.575 \times 10^{-3}$, $h_2 = 1.825 \times 10^{-3}$, $r_T = 4 \times 10^{-3}$

e) $\phi_0 = 723.810$, $h_1 = 0$, $h_2 = 2.5 \times 10^{-4}$, $r_T = 2.5 \times 10^{-3}$

f) $\phi_0 = 723.810$, $h_1 = 0$, $h_2 = 2.5 \times 10^{-4}$, $r_T = 4 \times 10^{-3}$

Parameter	Value
H	3.4mm
$h_2 - h_1$	2.5×10^{-4}m
r_o	1.1mm
D_1	1.6×10^{-9}m^2/s
D_2	4.85×10^{-9}m^2/s
V	0.057s^{-1}

Table 1: The parameter values used within the numerical simulations.

Figure 5

a) To produce the blue line:

$\phi_0 = 160, \qquad h_1 = 2.1531 \times 10^{-3}, \qquad h_2 = 2.4031 \times 10^{-3}, \qquad r_T = 2.5 \times 10^{-3}.$

To produce the red line:

$\phi_0 = 160, \qquad h_1 = 1.6881 \times 10^{-3}, \qquad h_2 = 1.9381 \times 10^{-3}, \qquad r_T = 2.5 \times 10^{-3}.$

b) To produce the blue line:

$\phi_0 = 160, \qquad h_1 = 2.2614 \times 10^{-3}, \qquad h_2 = 2.5114 \times 10^{-3}, \qquad r_T = 4 \times 10^{-3}.$

To produce the red line:

$\phi_0 = 160, \qquad h_1 = 2.2419 \times 10^{-3}, \qquad h_2 = 2.4919 \times 10^{-3}, \qquad r_T = 4 \times 10^{-3}.$

Figure 6

To create this plot, we solve the Partial Differential Equation (PDE) 101 times using equally spaced values of h_2 in the range

$$2.5 \times 10^{-4} \leq h_2 \leq 3.399 \times 10^{-3}.$$

The other parameters are chosen as follows:

$\phi_0 = 160, \qquad h_1 = h_2 - 2.5 \times 10^{-4}, \qquad H = 3.4 \times 10^{-3}, \qquad r_T = 2.5 \times 10^{-3}$

Figure 7

To create this plot, we solve the Partial Differential Equation (PDE) 101 times using equally spaced values of H in the range

$$5.01 \times 10^{-4} \leq H \leq 1.01 \times 10^{-2}.$$

The other parameters are chosen as follows:

$\phi_0 = 160, \qquad h_1 = h_2 - 2.5 \times 10^{-4}, \qquad h_2 = H - 1e - 6 \qquad r_T = 2.5 \times 10^{-3}$
