# Supplementary material for: Predicting physiologically-relevant oxygen concentrations in precision-cut liver slices using mathematical modelling
Source: PLoS One. 2022 Nov 2;17(11):e0275788. doi: 10.1371/journal.pone.0275788 (PMC9629643; doi:10.1371/journal.pone.0275788)
Supplement: Attachment — Submitted filename: Response to Reviewers PLOS ONE.docx [file pone.0275788.s002.docx]

Response to Reviewers

We wish to thank our reviewers for the exceptionally fast time in which they reviewed our manuscript, and for their constructive feedback. Please find our responses to the points raised below:

Reviewer 1:

1. Machine learning is a widely used tool in many different application areas but does not seem to have been used in many investigations relating to precision cut liver slices (PCLS) or similar. We have performed a wider literature search in this area and were only able to find one paper that uses machine learning (see below).

Abdeltawab H, Khalifa F, Hammouda K, Miller JM, Meki MM, Ou Q, et al. “Artifical Intelligence Based Framework to Quantify the Cardiomyocyte Structural
Integrity in Heart Slices”. Cardiovascular Engineering and Technology. 2022;13(1):170–180

This appears as reference [14] in the revised version of the manuscript and is discussed briefly in the penultimate paragraph of the Introduction.

2. The largest computational expense of our solution is solving the linear system of M equations given by (60) and (61), where $M\geq1$. As this system is solved using Gaussian elimination, the computational complexity of the solution is $O\left( M^{3} \right)$. We always choose $M\leq7$, so it takes Matlab approximately 1 second to compute and plot the solution. This information has been included in the section entitled “Numerical Implementation”, and a new reference relating to computational complexity has been provided as reference [18]. Details of the reference are below:

Arora S, Barak B. Computational complexity: a modern approach. Cambridge University Press; 2009

3. We have included some information about the limitations of our model and plans for future work in the “Conclusions” at the end of the paper. Our planned future work includes:

- Conducting experimental work on PCLS guided by the model derived in this work
- Solving a time-dependent model of oxygen diffusion and absorption to estimate how oxygen concentrations in PCLS change over time.

4. We agree that machine learning algorithms could prove to be a valuable tool in this area to help predict optimal positioning of a PCLS within a well to achieve physiologically realistic concentrations. However, the available literature in this area does not contain enough data to make the use of machine learning algorithms viable, and we do not currently have enough experimental data of our own to use either. We hope that this area will become increasingly data-rich over the coming years and make the use of these techniques feasible, but at the moment, the only possible mathematical models to use are deterministic, which is why we chose this particular approach.

As stated above in 1., we have included a small section about applications of machine learning in a similar area.

5. Please see 3. above for how we have responded to this point.

Reviewer 2:

No changes to the manuscript were requested, so we have no points to rebut. We thank you very much for your feedback and review.
